# Impact of human presence and activity on urban Eurasian red squirrels’ innovative problem-solving

**DOI:** 10.1093/beheco/araf104

**Published:** 2025-09-16

**Authors:** Pizza Ka Yee Chow, Olli J Loukola, Cwyn Solvi

**Affiliations:** Ecology and Genetics Research Unit, University of Oulu, Pentti Kaiteran katu 1, Linnanmaa, Oulu 90014, Finland; Division of Psychology, University of Chester, Parkgate Road, Chester, Cheshire CH1 4BJ, United Kingdom; Ecology and Genetics Research Unit, University of Oulu, Pentti Kaiteran katu 1, Linnanmaa, Oulu 90014, Finland; Guangdong-Hong Kong-Macao Greater Bay Area Center for Brain Science and Brain-Inspired Intelligence, Southern Medical University, No. 1023-1063 Shatai North Road, Guangzhou, Guangdong Province 510515, People’s Republic of China

**Keywords:** behavioral flexibility, human disturbance, leisure activity, technical innovation, urban ecology, wildlife cognition

## Abstract

Humans impact wildlife positively and negatively, and increasing evidence shows that humans potentially play a major role in shaping urban wildlife cognition. However, it remains unclear which, and how specific anthropogenic factors, shape animal cognitive performance. Here, across 15 urban areas in Oulu, Finland, we investigated how varied levels of human presence nearby, types of human activity (walking, dog-walking, cycling, and playground activities), and distance to the nearest footpaths influenced 64 squirrels’ innovative problem-solving ability—measured as the proportion of solving success at the site level, solving outcome at the individual level as well as individuals’ first-success latency. Higher mean human presence nearby and all measured human activities significantly decreased the proportion of success at the site level. Playground activity showed the highest negative impact on both the first- and subsequent-visit success rate at the site level. Increased mean human presence and walking decreased the likelihood of a squirrel successfully solving the novel food-extraction problem. When examining the problem-solving latency of individual squirrels, increased human presence also decreased squirrels’ first-success latency, and dog-walking was the outstanding factor affecting first-success latency. These results show the negative effects of specific human-related factors on an important cognitive trait, problem-solving ability. These factors may also potentially exert selective pressure on shaping urban wildlife cognition.

## Teaser

Humans potentially play a major role in shaping urban wildlife cognition. We examined how human presence, types of human activity, and distance to footpaths affected the innovative problem-solving performance of 64 free-ranging urban squirrels. Increased human presence negatively affected squirrels' solving success. Dog walking and playground activities had a greater impact than cycling on lowering squirrels' solving latency. These findings highlight that some human activities may potentially exert selective pressure on shaping urban wildlife cognition.

## Introduction

Urban environments have been shown to affect many aspects of wildlife, including physiology (Patankar et al. 2021), morphology (Evans et al. 2009; Biard et al. 2017), and behaviors (Sih et al. 2011; Candolin and Wong 2012; Lowry et al. 2012; Sih 2013; Ritzel and Gallo 2020; Caspi et al. 2022). A recent growing body of evidence suggests that the impacts of urbanization can extend to cognition (Ducatez et al. 2015; Papp et al. 2015; Chow et al. 2021; 2024). Cognition manifests through behaviors, and can be defined as an individual acquiring, processing, storing, utilizing, and reacting to information about their environment (Shettleworth 2010). Cognition can enable wildlife to adapt their behaviors to meet environmental challenges (Lee and Thornton 2021; Sarkar and Bhadra 2022). In urban environments, where humans are a dominant feature (Gross 2016), human presence and activity are expected to induce significant selective pressures on various aspects of wildlife (eg, morphology, behavior and cognitive traits, see review by Lerman et al. 2020; Schell et al. 2021; Sarkar and Bhadra 2022). However, which characteristics of human activity affect wildlife cognitive performance remains largely unclear, even though such investigations are crucial for sustainable urban planning and city management, particularly in designing green spaces for recreational use (Cardona et al. 2024).

Human activities may provide opportunities for wildlife to consume anthropogenic or novel food via humans directly feeding animals or indirectly from feeding stations, waste bins, and food left behind from human gatherings (eg, Belant 1997; Murray et al. 2015; Demeny et al. 2019; Burt et al. 2021; Griffin and Ciuti 2023; Rimbach et al. 2023), thus increasing the likelihood of wildlife settling in urban habitats (Goumas et al. 2020; Wilson et al. 2020). However, human activities can often alter the behavior of urban wildlife (Blanc et al. 2006). Wildlife's behavior can be affected by the characteristics of human activities, such as the type of activities, the number of humans present in an area, the distance from potential threats, and the speed of the threat stimulus (Blanc et al. 2006; Bateman and Fleming 2014; Tablado and Jenni 2017; Burton et al. 2024; Ramellini et al. 2024). For example, urban hooded crows (*Corvus cornix*), Eurasian robins (*Erithacus rubecula*), and wood pigeons (*Columba palumbus*) adjust their activity patterns depending on the number of humans in a park (Ramellini et al. 2024), and Eastern gray squirrels (*Sciurus carolinensis*) show higher alertness in areas with high human activity, particularly where dogs are present (Cooper et al. 2008). Adverse impacts can result from common, seemingly harmless activities, such as walking, dog-walking, and cycling. Disturbance may cause both short- and long-term effects on wildlife activities, leading them to adjust their spatial usage and temporal activity patterns (Gaynor et al. 2018; Burt et al. 2021; Doherty et al. 2021), which can have fitness consequences (Bötsch et al. 2017). Despite this, investigations on the impacts of various human activities on wildlife behavior and, in particular, cognition remain limited.

Investigations into urban wildlife cognition are still in the early stages (Lee and Thornton 2021), but a few human-related factors have already been shown to affect wildlife cognition, and in particular innovation (ie, innovative problem-solving). Innovation relies on cognition (Ducatez et al. 2015) and can be assessed using an artificial food-extraction paradigm (Griffin and Guez 2014), where individuals use existing behaviors to overcome obstacles to obtain food rewards in novel situations (eg, feeders or puzzle boxes) (Kummer and Goodall 1985; Reader and Laland 2003). Thus far, the few studies that have examined the effects of specific human-related factors on innovation indicate that the presence of a human can directly affect the ability of wild-caught captive juvenile house finches (*Haemorhous mexicanus*) to extract food from a novel artificial feeder (Cook et al. 2017) and that the number of humans present in a site directly affects group/site-level solving success and individuals’ solving latency in wild Eurasian red squirrels (*Sciurus vulgaris*) (Chow et al. 2021). Human presence has also been shown to indirectly affect other cognitive abilities, such as generalization or the capacity to apply learned information to solve similar problems (Chow et al. 2024). However, more focus is needed on comparing the diverse human-related factors. Such research can identify specific features of human activity that shape urban wildlife cognition, which can help to identify the traits and mechanisms that enable urban wildlife to adapt to or thrive in urban environments.

Here, our goal was to examine how different aspects of human activity affect the innovative problem-solving performance of urban Eurasian red squirrels. The ability to innovate, such as extracting food from a bird feeder, is likely related to the fitness of urban squirrels (Krauze-Gryz and Gryz 2015; Kostrzewa and Krauze-Gryz 2020). Red squirrels demonstrate a remarkable ability to adapt to varying urban environments (Babińska-Werka and Żółw 2008; Uchida et al. 2016; Jokimäki et al. 2017; Thomas et al. 2018; Shimamoto et al. 2020) and take advantage of living alongside humans (Fingland et al. 2022; Wist et al. 2022). They are opportunistic foragers and food and habitat generalists (Reher et al. 2016; Thomas et al. 2018); they consume anthropogenic food and extract food from novel artificial problems (eg, Chow et al. 2018, 2021). We used a food-extraction task (hereafter, the puzzle box) that was previously established and used to assess innovative problem-solving performance in red squirrels from different populations residing in different countries (Chow et al. 2018, 2021). These previous works were in sites where humans and squirrels had minimal direct interactions (eg, hand feeding) and showed that increased exposure to humans led to fewer urban red squirrels succeeding at solving the task (group/site level) but caused those squirrels that did succeed to have faster solving times over successes (individual level). However, it remains unclear what types of human-related factors (eg, types of activities) influence squirrels’ innovative problem-solving performance. To address this gap, we conducted a field experiment with urban red squirrels residing in areas where humans and squirrels had minimal direct interaction and related their innovative problem-solving performance to 3 key aspects of human-activity factors: number of humans present, type of human activity, and distance to the nearest footpath.

Our general hypothesis was that the 3 aspects of human-activity factors would affect squirrels’ innovative problem-solving performance, both at the site and individual level. Previous findings (eg, Chow et al. 2021) suggested the prediction that all three factors would negatively relate to innovative problem-solving performance; an increase in human activity would decrease successful solving and lead to a lower first-success latency. Below, we outline the rationale for the predictions for each specific variable:

1. *Number of humans present.* The number of humans present in an area reflects the level of activity intensity in that area. We followed previous research (Chow et al. 2021) and predicted that an increased number of humans in a site (ie, higher activity intensity) would decrease solving success and solving latency (increase efficiency).
2. *Types of human recreational and leisure activity.* This variable captures characteristics of human activity at a more granular level. Previous findings have shown that urban red squirrels initiate flight responses to approaching humans or dogs (Uchida et al. 2019), suggesting that walking or dog-walking would decrease solving success and solving efficiency. We further included other common human activities in urban areas, cycling and playground activities, to compare the varied impacts on solving performance, and expected these 2 human activities would have a similar impact on solving performance, that is, cycling and playground activities would lower the success rate and first-success latency.
3. *Distance to the nearest footpath.* Footpaths are designated areas for human activity. Previous studies have shown that close proximity to these paths can influence wildlife behavior, for example, increased vigilance in elks (*Cervus elaphus*) and flushing behavior in vesper sparrows (*Pooecetes gramineus*) and western meadowlarks (*Sturnella neglecta*) (Miller and Knight 2001; Ciuti et al. 2012). We therefore predicted that a shorter distance to a footpath would decrease squirrels’ innovative problem-solving success rate and first-success latency due to increased exposure to a perceived threat or stressor.

## Materials and methods

### Study sites

Between August and November 2021, we conducted a field experiment in 15 green spaces (‘sites’) in Oulu, Finland (see map on Google Earth, Table S1 for site details). Sites are a district or a quarter of a district that have developed green spaces (a mix of fragmented urban forests and urban parks) in residential and business areas defined by the city management of Oulu (NLS Finland) and via Google Maps. The sites primarily contained sections with a mix of deciduous and coniferous trees, including silver birch (*Betula pendula*), downy birch (*B. pubescens*), Norway spruce (*Picea abies*), and Scots pine (*Pinus sylvestris*), creating a blend of natural habitat providing safety and food sources for red squirrels (Rubino et al. 2012). The sites were mostly flat and were traversed by footpaths designated by city management. Human activities varied in these sites, and the frequency of using different footpaths also varied. In all sites, there were trampling impacts on the ground, indicating humans (and their dogs) frequently traversed areas amongst the trees and bushes off the main footpaths. According to the Finnish Public Order Act (Järjestyslaki 27.6.2003/612, Section 14), dogs must be on leash in public areas. Despite this, our observations indicate that dogs are frequently unleashed (70.6% of all observed humans with dogs across sites). There was an average of 4 squirrels foraging in each site (ranging from 3 to 6; Table S1), which is in line with the previously reported squirrel population density in fragmented urban green areas in Finland (Jokimäki et al. 2017). Most sites were 400 to 500 m apart (with around 300 m apart in one occasion between 2 sites), which is the average movement of urban squirrels (Andrén and Delin 1994). Throughout the experiment, we never saw the same individual at more than one site (and therefore no pseudo-replication), likely because of the high fragmentation of the urban habitats restricting squirrels’ movements (Thomas et al. 2018). Individual identity was confirmed via video recordings (see below and Note S2 for details). We did not observe any feeders for squirrels or humans feeding squirrels. The following human activities were observed and used for analyses: walking, dog-walking, playground activities, and cycling and activity levels varied across sites (Table S1).

The field experiment was conducted in multiple sites (2 to 4 sites) simultaneously and daily to control for any weather and weekend/weekday effects. In each site, we placed one puzzle box in a location that was safe for squirrels for the experiment (eg, close to a tree, under a tree canopy, and away from major roads); this aimed to minimize potential fatalities (Fingland et al. 2022). The distance between the box location at each site and the nearest footpath varied (15 to 79 m, see Table S1). We attached a trail camera (Browning model no: BTC-7E-HP4) to a tree to monitor squirrels’ task performance and for individual identification. Individual identification followed the protocol employed by Chow and colleagues (Chow et al. 2018, 2021); we identified each individual using their unique characteristics (eg, facial features, fur color, and tail shapes) from video footage (see Note S2). This process involved an initial intensive frame-by-frame analysis of video footage to identify each squirrel, followed by reanalyzing the footage 3 to 5 mo after the initial analysis to obtain intra-rater reliability (Cohen's Kappa intra-reliability result = 0.99).

### Puzzle box

**Fig. 1.**
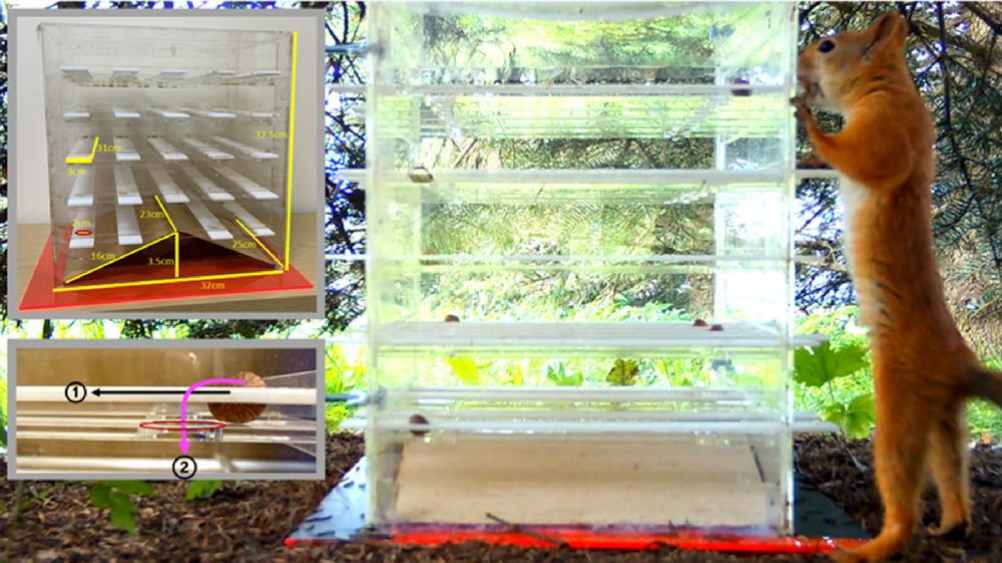
The puzzle box required innovation from squirrels to obtain a highly desired food (hazelnuts). The main image shows a squirrel (Bis Bis) attempting to solve the puzzle box used in this study (Dimensions shown in upper left inset). A squirrel had to either push a lever if the hole was on the squirrel's side of the box, or pull a lever if the hole was on the other side of the box. These pull-push actions would dislodge the lever or cause 2 holes (one in the lever and one in an immobile plank under the lever) to align and allow a nut (if there is a nut in the hole) to fall through and out of the box (inset bottom left, 1-2).

The puzzle box presented to squirrels was adapted from an established task that has been solved by ∼ 60% of red squirrels from different populations (Chow et al. 2018, 2021), thus making it a suitable task to reveal variations of performance for this species. We adapted the task by increasing the number of levers (and nuts) in the box to help reduce how often we needed to visit/check each site (ie less refilling was required which minimized experimenter disturbance). The puzzle box was made of transparent acrylic sheets (shown in Fig. 1 and inserts with dimensions). It contained nuts (hazelnut kernels) each positioned within a hole of a lever on top of an immobile plank. Only half of the levers (12 of 24) contained a nut. Squirrels could see and smell the nuts through the box given the various small gaps but could not directly reach the nuts. To solve the task, a squirrel had to either push a lever if the hole was on the squirrel's side of the box, or pull a lever if the hole was on the other side of the box (Fig. 1a, Video S1). These actions would dislodge a lever or cause the hole in the lever to align with a hole in the plank, thus allowing the nut (if there was a nut in the hole) to fall, hit the slanted floor, and roll out from the box from one of the side openings (22.5 cm × 3 cm). These 2 scenarios were considered successful to account for various motivations (eg, squirrels may explore the apparatus in a variety of ways, including pulling and pushing levers, as a result of hunger or play).

### Procedures

The whole experiment in each location lasted for an average of 18 d, and followed a standardized procedure. Before the puzzle box was presented to squirrels, we pre-baited the selected location twice a day in each site using unshelled hazelnuts; this aimed to attract squirrels foraging in the site to regularly visit the location. During the pre-baiting period, we started observing the varied human traffic and activity at each site (see “Measurements” section below). A trail camera was mounted to a tree, around 1 m away from the box to capture squirrels’ visitation; this allowed us to identify individuals using their characteristics as well as behavior throughout the experiment. Once the same squirrels began visiting and obtaining nuts from the location regularly (daily), we carried out a habituation phase. In this phase, we presented the box without levers to squirrels for 4 to 5 h and placed hazelnuts around the puzzle box; this phase aimed to minimize any neophobic response to the box in the main experimental phase. When the squirrels obtained all the nuts around the box, we started the main experiment in which levers were inserted into the box. The squirrels were free to come and leave the puzzle box, which ensured their motivation for engaging with the experiment was high. In the main experimental phase, data was collected simultaneously in multiple sites daily from dawn to noon (for an average of 10 consecutive days per site, ranging from 8 to 12 d), during squirrels’ most active time regardless of weather conditions. The data collection period and specific procedures (see below) helped minimize confounding variables, such as weather, weekend/weekday effect on human activity, daylight availability and kept a consistent high level of squirrel participation in the experiment. During the experiment, we checked and refilled box 3 to 4 times per day. Each check was conducted at a similar time of the day across sites, and the inter-check/observation interval was between 45 min and 1.5 h. These procedures were designed to obtain consistency in observing the level of human activity and minimize potential social interference among squirrels (less than 2% of the video clips had 2 or more squirrels). In each check, we randomized the facing direction of the box, the levers that contained a nut, and the facing direction of the levers.

## Measurements

### Human-activity factors

Human-activity data were obtained at similar times at multiple sites, and adjusted to seasonal time changes. Our measurements of human-activity factors included the following:

1. Human presence nearby was defined as the number of humans within 100 m (in radius) around the box, which reflects the disturbance threshold distance noted for small mammals (Dertien et al. 2021). Four to five observations were made per day, including before setting up the box, during each check of the box (3 to 4 per day), and at the end of the field experiment for the day. Each observation entailed counting the number of humans in the immediate area around the puzzle box location in a 5-min period. For each site, we obtained the mean number of humans around the puzzle box location per observation for analysis, which was the total number of humans around the box across all observations divided by the number of observations made at that site throughout the experiment.
2. Types of human activity were noted by the experimenter during the 5-min observation. The types of activities were later put into one of the following five categories: “walking”, “dog-walking”, “cycling”, “playground activities” (eg, gathering, playing in the playground, ball games such as football), and “other activities” (eg, construction work, bin collection, grass cutting). During each 5-min observation, the number of humans engaged in that activity was recorded, which was used to determine the frequency of that activity. To calculate the mean frequency for each activity per observation, we divided the total number of humans participating in each activity by the total number of observations across the field experiment days at each site. This approach allowed us to estimate the intensity of human presence with a more specific focus on the type of activity, in contrast to the general human presence calculated previously.
3. Distance to the nearest footpath was measured as the shortest distance (in meters) between the nearest footpath and the location where we placed the puzzle box. This was determined using the measurement tools of Google Maps.

### Innovative problem-solving performance

The innovative problem-solving performance of the squirrels was assessed following (Chow et al. 2021); whenever a squirrel manipulated a lever using its paws, teeth or nose, we obtained 2 measurements until the squirrel ceased its attempts. The first measurement was “solving outcome”, which indicated whether the manipulation was successful, ie dislodged a level or caused a nut aligned with the hole in a lever with the hole in a plank. The second measurement was “solving latency”, defined as the time spent manipulating the lever prior to a successful outcome. In line with previous studies (Chow et al. 2021), squirrels were classified based on the solving outcome: those who solved the problem multiple times were categorized as “innovators” and those who only succeeded once but could not replicate the success or failed to solve the problem throughout the presentation period were “non-innovators”. Among the innovators, we further considered squirrels that solved the problem on their first visit as “first-visit solvers” and those that solved the problem on subsequent visits as “subsequent solvers”. Aside from the individual solving outcome, we also obtained site-level performance, for which we counted the number of squirrels that successfully solved the problem at a site and divided this number by the total number of squirrels that interacted with the puzzle box at that site; this reveals the effect of human-activity factors on solving performance at a site level. Only squirrels that had successfully solved the problem provided data for solving latency. For each solver, we summed across all the durations of failed manipulations until a success occurred to obtain the first-success latency.

#### Data analyses

All data were analyzed using R version 4.4.1 (The Comprehensive R Archive Network). Before running the models, we first checked the correlations among variables of interest using Pearson's *r* (see Tables S3–S6). To avoid multicollinearity that would induce interpretation problems (Allen 1997), we excluded variables that have a moderate to high correlation *r* ≥ 0.5 (walking and cycling: *r* = 0.95, Table S4) in the same model when examining human activities on solving performance (Dancey and Reidy 2007), but we ran separated models (ie, including only one of the variables) to fully test the hypotheses (see Table S2 for model specification). After each model was run, we used the Variance Inflation Factor (VIF) in the “car” package (Fox et al. 2012) to further assess multicollinearity (VIF < 5, tolerance >0.2) (see results below). All results reported here are 2-tailed and the significance level was set at *P* ≤ 0.05.

The proportion of successes at the site level was analyzed using beta regression of the “betareg” package (Cribari-Neto and Zeileis 2010). Individuals’ solving outcome and (first-success) solving latency were analyzed using a Generalized Linear Mixed Model (GLMM) with binomial logit link and gamma log-link distribution in the “glmmTMB” package (Magnusson et al. 2017), respectively; the use of gamma log-link distribution can accommodate the skewed distribution. Site was the random variable in the individual analyses (see details of model in Table S2).

We first examined the factors that affected the innovative problem-solving performance of first-visit solvers. Three main models included the fixed effects of distance (m) to the nearest footpath and the mean number of humans nearby whereas the proportion of successes at the site level was the response variable in one model (*N* = 15, Table S2 Model 1a), individual outcome in another model (*N* = 64, Table S2 Model 1c), and individuals’ first-success latency was in the final model (*N* = 43, Table S2 Model 1d). In all models, we also included squirrel group size (the number of squirrels that participated in the experiment at each site) as another fixed effect because this variable has been shown to affect solving performance (Chow et al. 2021). We reran the models by including the subsequent solvers to examine how these factors affected the innovative problem-solving performance of all innovators at the site (*N* = 15, Table S2 Model 1b) and individual level (*N* = 53, Table S2 Model 1e).

We then examined whether the types of human activity and distance to the nearest footpath affected each problem-solving performance measure that included: (1) success rate at the first-visit at the site level (*N* = 15); (2) success rate at the first and subsequent visit at the site level (*N* = 15); (3) individuals’ solving outcome (*N* = 64); (4) first-visit solvers’ first-success latency (*N* = 43); and (5) all innovators’ first-success latency (*N* = 53) (Table S2, Models 2 to 3). Due to the high correlation between walking and cycling (*r* = 0.94 and *r* = 0.95) (see Table S4), we ran 2 separate models for each measure to fully examine the hypothesis. One model included fixed factors of walking, dog-walking, playground activities and distance of nearest footpath. The other model included fixed factors of cycling, dog-walking, playground activities and distance of nearest footpath. In all the models, variables were standardized to compare their effect size.

## Results

### Human presence nearby, distance to the nearest footpath, squirrel group size and solving success

**Fig. 2.**
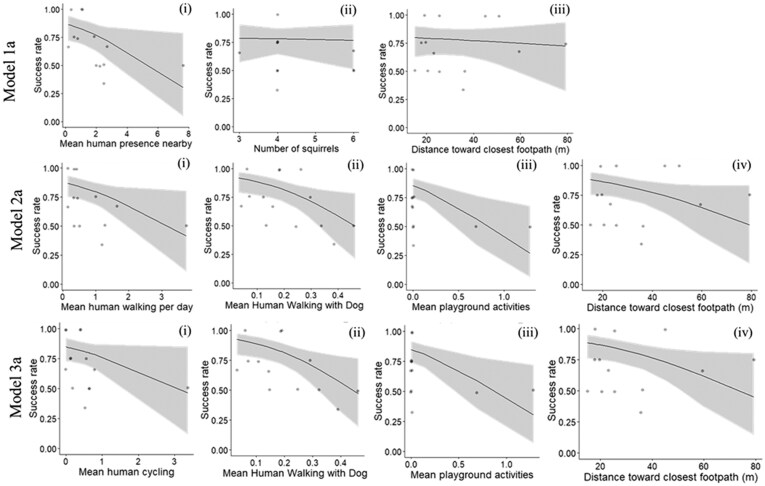
Site-level analysis. Model 1a shows the effects of (i) mean human presence nearby, (ii) squirrel group size, and (iii) distance to the nearest footpath (m) on the first-visit success at the site level (*N* = 15). Models 2a and 3a show the effects of different types of human activities (i to iii) and distance to the nearest footpath (iv) on the first-visit success rate at the site level (*N* = 15).

In the selected 15 sites, a total of 64 squirrels participated in the puzzle box task. Fifty-three (83%) squirrels were innovators, and 43 (68%) squirrels solved the problem on their first visit (ie, first-visit solvers) and another 10 squirrels solved the problem on a subsequent visit (ie, subsequent solvers). At the site level, human presence nearby, distance to the nearest footpath and squirrel group size explained 18% of the variance in the proportion of first-visit success (*N* = 15, Table 1 Model 1a, Fig. 2 Model 1a). First-visit success rate significantly decreased with an increased mean human presence nearby (Z = −1.98, *P* = 0.048) (Fig. 2 Model 1ai). However, neither squirrel group size (Z = −0.13, *P* = 0.894) (Fig. 2 Model 1aii) nor distance to the nearest footpath (Z = −0.37, *P* = 0.710) (Fig. 2 Model 1aiii) affected the first-visit success rate. The other model including all innovators (first-visit or subsequent-visit solvers) showed that human presence nearby, distance to the nearest footpath and squirrel group size explained 29% of the variance in the proportion of successes at the site level (*N* = 15, Table 1 Model 1b), and these factors explained 18% of variance in individual solving outcome (*N* = 64, Table 1 Model 1c). However, similar to the model of the first-visit success, mean human presence nearby was the only factor that significantly negatively affected the overall success rate at the site level (Z = −2.60, *P* = 0.009) and individual level (Z = −2.14, *P* = 0.032).

**Table 1.** Models (M) predict the proportion of success at the site level (*N* = 15) of (a) the first-visit solvers, (b) first and subsequent solvers and individual level (*N* = 64), and (c) solving outcome at individual level on first and subsequent visits.

		Response variables														
		(a) Site level (*N* = 15): Proportion of first-visit success (first-visit solvers)					(b) Site level (*N* = 15): Proportion of first and subsequent visit success (all solvers)					(c) Individual level (*N* = 64): solving outcome of first and subsequent visits (all solvers)				
M	Fixed factors	Est	S.E.	Z	P	MR^2^	Est	S.E.	Z	P	MR^2^	Est	S.E.	Z	P	MR^2^
**1**	Squirrel group size	−0.04	0.32	−0.13	0.894	0.18	0.06	0.27	0.20	0.838	0.29	0.08	0.54	0.15	0.878	0.18
	Mean number of humans present per observation	−0.66	0.33	−1.98	**0**.**048**	…	−0.43	0.17	−2.60	**0**.**009**	…	−0.88	0.41	−2.14	**0**.**032**	…
	Distance of nearest footpath	−0.12	0.32	−0.37	0.71	…	−0.01	0.02	−0.87	0.385	…	−0.31	0.45	−0.70	0.483	…
**2**	Walking	−0.58	0.29	−2.02	**0**.**043**	0.29	−0.71	0.25	−2.79	**0**.**005**	0.49	−1.20	0.51	−2.37	**0**.**018**	0.62
	Dog walking	−0.71	0.31	−2.25	**0**.**024**	…	−0.37	0.29	−1.28	0.200	…	−1.52	0.9	−1.69	0.090	…
	Playground activities	−0.79	0.29	−2.71	**0**.**007**	…	−0.80	0.26	−3.01	**0**.**002**	…	−1.52	0.84	−1.81	0.070	…
	Distance of nearest footpath	−0.58	0.32	−1.83	0.067	…	−0.49	0.30	−1.65	0.099	…	−1.71	0.99	−1.73	0.084	…
**3**	Cycling	−0.46	0.28	−1.67	0.095	0.25	−0.59	0.25	−2.38	**0**.**017**	0.44	−0.93	0.38	−2.47	**0**.**014**	0.52
	Dog walking	−0.76	0.32	−2.4	**0**.**017**	…	−0.42	0.29	−1.45	0.148	…	−1.29	0.74	−1.75	0.080	…
	Playground activities	−0.72	0.29	−2.49	**0**.**013**	…	−0.72	0.26	−2.73	**0**.**006**	…	−1.06	0.61	−1.75	0.080	…
	Distance of nearest footpath	−0.66	0.32	−2.05	**0**.**041**	…	−0.59	0.30	−1.95	0.051	…	−1.38	0.78	−1.77	0.077	…

Model 1 examined general factors that included squirrel group size, mean number of human presence nearby and distance to the nearest footpath as fixed factors. Models 2 and 3 emphasized the effects of the types of human activities on innovative problem-solving success at the site level and solving outcome at the individual level, respectively. Due to the high correlation between walking and cycling (Table S4), separate models were run to examine the predictions fully. Model 2 included walking, dog walking, playground activities and distance to the nearest footpath as fixed factors. Model 3 included cycling, dog walking, playground activities and distance to the nearest footpath as fixed factors. This table shows standardized estimate (Est), standard error (S. E.), Z and *P* values, and Marginal R (MR^2^). Bold values indicate P < 0.05.

### Types of human activity, distance to the nearest footpath, and solving success

The most common human activity across the sites was walking (37.1%), followed by cycling (26.4%), dog-walking (16.8%), other activities (5.6%) and playground activities (3.7%) (see Table S1 for human activity by site). For site-level analysis, the model that included walking, dog-walking, playground activities and distance to the nearest footpath explained 29% of the variance in the proportion of first-visit success at the site level (*N* = 15, Table 1 Model 2a). Except for the distance to the nearest footpath, all types of human activity significantly decreased the proportion of first-visit success at the site level (*N* = 15, Table 1 Model 2a, Fig. 2 Model 2a). Playground activities had the highest effect size (−0.79) followed by dog-walking (−0.71) and walking (−0.58). Another site-level analysis that included cycling, dog-walking, playground activities and distance to the nearest footpath for first-visit solvers explained 25% of the variance in the proportion of first-visit success at the site level (*N* = 15, Table 1 Model 3a). Except for cycling, all the variables significantly decreased the proportion of first-visit success (Table 1 Model 3a, Fig. 2 Model 3a). Dog-walking (−0.76) had a slightly higher effect size than playground activities (−0.72).

When including first and subsequent solvers in the model, the model that included walking, dog-walking, playground activities and distance to the nearest footpath explained 49% of the variance in the proportion of success at the site level (*N* = 15, Table 1 Model 2b). Walking and playground activities significantly decreased the proportion of success at the site level, and playground activities had the highest effect size (−0.80) followed by walking (−0.71) (Table 1 Model 2b). Another site-level analysis included cycling, dog-walking, playground activities and distance to the nearest footpath for first-visit solvers explained 44% of the variance in the proportion of success at the site level (*N* = 15, Table 1 Model 3b). Cycling and playground activities significantly decreased the proportion of success at the site level, and playground activities had the highest effect size (−0.72) followed by cycling (−0.59) (Table 1 Model 3b).

For the individual-level analysis, the model that included walking, dog-walking, playground activities and distance to the nearest footpath explained 62% of individual solving outcome (*N* = 64, Table 1 Model 2c), and only walking significantly decreased the likelihood of succeeding in solving the novel problem at the individual level (Table 1 Model 2c). The model that included cycling, dog-walking, playground activities and distance to the nearest footpath explained 52% in the solving outcome, and only walking significantly decreased the likelihood of succeeding in solving the novel problem at the individual level (Table 1 Model 3c).

### Human presence nearby, distance to nearest footpath and first-success latency

**Fig. 3.**
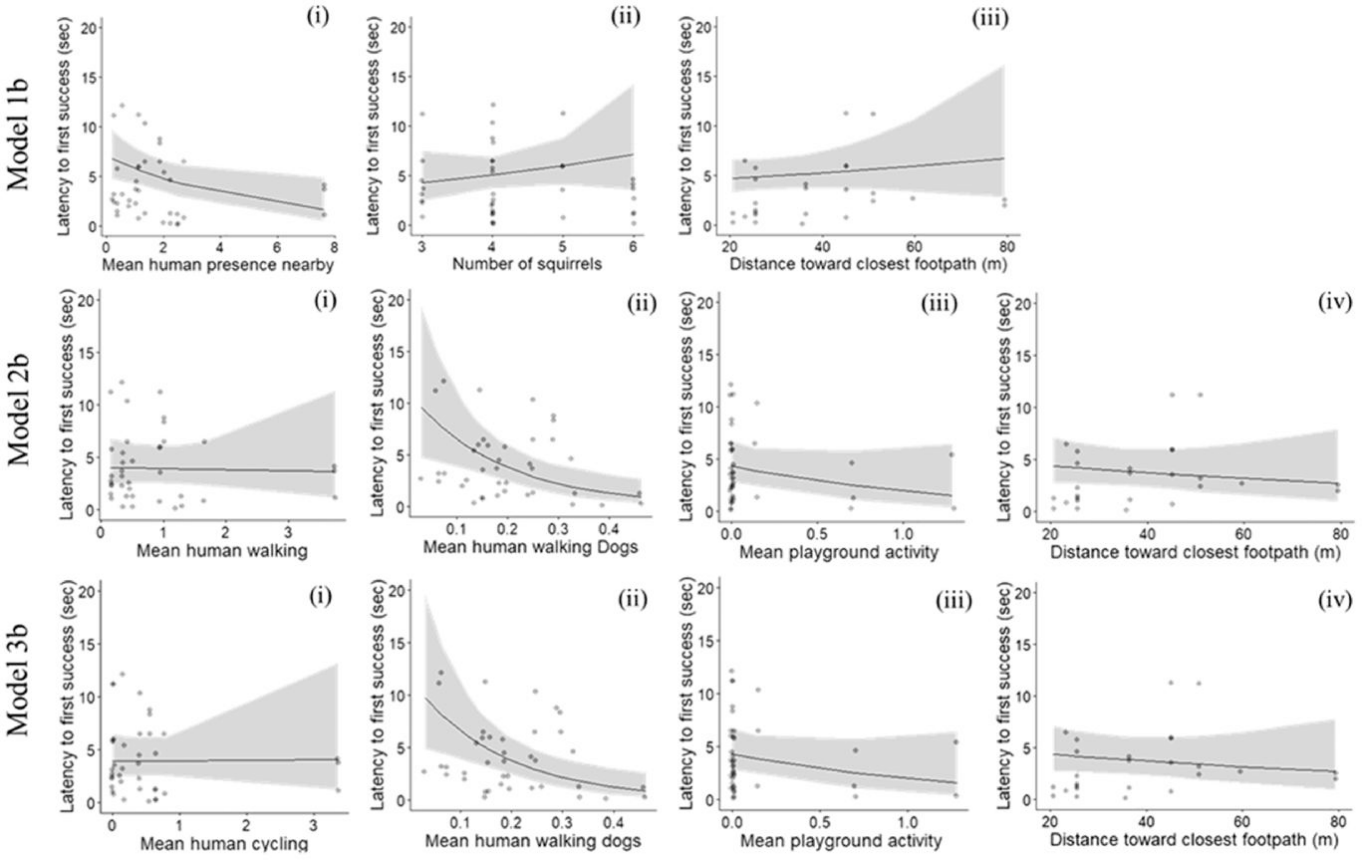
Individual-level analysis. Model 1b shows the effects of (i) mean human presence nearby, (ii) squirrel group size, and (iii) distance to the nearest footpath (m) on the first-success latency of first-visit solvers (*N* = 43). Models 2b and 3b examined the effects of different types of human activities (i to iii) and distance to the nearest footpath (iv) on first-visit solvers’ first-success latency (*N* = 43).

First-visit solvers (*N* = 43) took around 6 s (SD = 7.01) to cause a nut to fall or to dislodge an empty lever (see Panda, an innovator in the Video S1). For these first-visit solvers, the model that included squirrel group size, mean human presence nearby, and distance to the nearest footpath (m) explained 17% of the variance in first-success latency (Table 2 Model 1a, Fig. 3 Model 1b). Only human presence nearby significantly affected these innovators’ first-success latency (Z = −2.21, P = 0.041); increased mean human presence nearby decreased their first-success latency (Fig. 3 Model 1bi). Ten more squirrels subsequently solved the problem, with a mean solving latency of 8 s (SD = 9.50, *N* = 53). Squirrel group size, mean human presence nearby, and the distance to the nearest footpath explained 17% of the variance in their first-success latency (*N* = 53, Table 2 Model 1b); shorter first-success latency was associated with a larger squirrel group size (Z = 1.98, *P* = 0.048) and a higher mean human presence nearby (Z = −2.20, *P* = 0.028). Distance to the nearest footpath did not affect the first-success latency (Z = −0.11, *P* = 0.916).

**Table 2.** Models (M) included fixed factors of human activities and distance of nearest footpath to predict individual first-success latency of (a) first-visit solvers (*N* = 43) and (b) all solvers (*N* = 53).

		Response variables									
		(a) first-success latency for first-visit solvers (*N* = 43)					(b) first-success latency for all solvers (*N* = 53)				
M	Fixed factors	Est	S.E.	Z	P	MR^2^	Est	S.E.	Z	P	MR^2^
**1**	Squirrel group size	0.18	0.21	0.86	0.390	0.17	0.31	0.16	1.98	**0**.**048**	0.17
	Mean number of humans per observation	−0.38	0.19	−2.05	**0**.**041**		−0.42	0.19	−2.20	**0**.**028**	
	Distance of nearest footpath	0.12	0.19	0.61	0.541		−0.02	0.15	−0.11	0.916	
**2**	Walking	−0.10	0.18	−0.59	0.554	0.37	−0.22	0.19	−1.14	0.256	0.20
	Dog walking	−0.60	0.21	−2.84	**0**.**005**		−0.21	0.16	−1.29	0.196	
	Playground activities	−0.22	0.18	−1.28	0.199		0.20	0.16	1.26	0.208	
	Distance of nearest footpath	−0.06	0.15	−0.38	0.706		0.14	0.16	0.85	0.397	
**3**	Cycling	−0.06	0.18	−0.36	0.721	0.37	−0.19	0.20	−0.96	0.338	0.20
	Dog walking	−0.62	0.21	−2.94	**0**.**003**		−0.23	0.16	−1.44	0.151	
	Playground activities	−0.21	0.17	−1.20	0.230		0.24	0.16	1.51	0.130	
	Distance of nearest footpath	−0.06	0.15	−0.42	0.676		0.14	0.16	0.83	0.404	

Model 1 examined general factors that included squirrel group size, mean number of humans present nearby and distance to the nearest footpath as fixed factors. Models 2 and 3 emphasized the effects of the types of human activities on innovative problem-solving success at the site level. Model 2 included walking, dog walking, playground activities and distance to the nearest footpath as fixed factors. Model 3 included cycling, dog-walking, playground activities and distance to the nearest footpath as fixed factors. This table shows standardized estimate (Est), standard error (S. E.), *Z* and *P* values, and Marginal R (MR^2^). Bold values indicate *P* < 0.05.

### Types of human activity, distance to the nearest footpath, and solving latency

For the first-time solvers (*N* = 43), the model that included walking, dog-walking, playground activities, and the distance to the nearest footpath explained 37% of the variance in the first-success latency (Table 2 Model 2a). Only dog-walking significantly affected their first-success latency (Z = −2.84, *P* = 0.005) (Table 2 Model 2a, Fig. 3 Model 2bii). The model that included cycling, dog-walking, playground activities and distance to the nearest footpath also explained 37% of the variance in the first-success latency (Table 2 Model 3a). Similar to the previous model, only dog-walking affected first-success latency (Z = −2.94, *P* = 0.003); more dog-walking led to shorter first-success latency (Fig. 3 Model 3bii). For both first and subsequent solvers (*N* = 53), both models explained 20% of the variance in first-success latency and none of the activities nor distance to the nearest footpath significantly affected first-success latency (Table 2 Model 2b & Model 3b).

## Discussion

Here, we provided new evidence on how different aspects of human activity affect innovative problem-solving performance. Key findings include the following: (1) An increased mean human presence nearby negatively affected the proportion of success both at the site level and individual level as well as individuals’ first-success latency. (2) All types of human activities (walking, dog--walking, cycling, and playground activity) significantly decreased the (first and/or subsequent visit) the proportion of success at the site level, whereas only walking decreased the likelihood of solving success at the individual level. (3) Playground activity had the highest negative impacts on the proportion of success at the site level. However, walking was the factor that consistently affected the proportion of success both at the site and individual level. (4) First-visit solvers were quicker to succeed if dog-walking occurred nearby. These results highlight key aspects of human activity affecting squirrels’ innovative problem-solving ability, a trait that is potentially important for successful settlement in urban environments (Barrett et al. 2019).

The negative impacts of increased human presence on innovative problem-solving performance support previous studies that suggest that human presence and activity are stressors for urban squirrels and that squirrels perceive humans as potential threats (Uchida et al. 2016). In this study, the squirrels had minimal interaction with humans and solving the innovation task required them to change from their typical foraging pattern in trees to the ground (Shuttleworth 2000). Nearby humans may cause some squirrels to fail or abandon the task after the first and subsequent visits. However, those who successfully solved the problem showed a shorter first-success latency with increased human presence in a site. The enhanced solving efficiency in response to increased human presence may be considered as an adaptive strategy, allowing squirrels to quickly retreat to a tree for safety (Chow et al. 2024).

In urban areas, 4 common types of human activities (walking, dog-walking, playground activities, and cycling) likely influence squirrels’ behavior in response to human disturbance (Bateman and Fleming 2014; Uchida et al. 2019; Uchida et al. 2020). We sought to determine which specific types of human activities are most impactful. Our results support the prediction that human activities like walking and dog-walking would decrease the proportion of success at the site level and individuals’ first-success latency. These results may be explained by the fact that humans (and their dogs) moved freely in our study sites, especially since dogs were often not on leash (as per our observations) and their movement can be highly unpredictable, eg, going off designated footpaths, chasing or approaching squirrels (Weston and Stankowich 2014). Importantly, walking was the only significant factor to affect the proportion of success both at the site and individual levels, highlighting the negative impact of this human activity on solving novel problems, a means by which individuals can utilize alternative food sources in urban habitats.

In urban environments, dogs are common predators of squirrels (Wauters et al. 1997; Makowska and Kramer 2007). Dog-walking, especially when the dog is unleashed, may lead to various adverse effects on wildlife, including injury or death (Weston and Stankowich 2014). Given that urban squirrels have a high overlapping active time with domestic dogs (Tobajas et al. 2023), urban squirrels often remain vigilant, as part of the risk assessment of their environment (Blumstein 2003), in response to human walking and walking with dogs (Cooper et al. 2008; Uchida et al. 2019, also see Video S2).

The fact that, in our study, dog-walking was linked to a faster solving latency for first-visit solvers suggests that frequent encounters with dogs may shape urban squirrels’ cognition, for example, favoring individuals that can detect risk faster and complete tasks swiftly, which has potential consequences for their energy budgets, survival, and settlement in urban environments.

Both cycling and playground activity only negatively impacted (first and/or subsequent visit) success rate, which partially support our prediction. The travel speed of cyclists can be unpredictable (Taylor and Knight 2003), and playground activity often induces loud noises of varied duration (Rosenthal et al. 2022). Characteristics like these may trigger anti-predator responses (McRae 2020), leading to failed attempts at problem-solving. Compared with cycling, playground activity had a higher negative impact on problem-solving success rate. Notably, playground activity had the fewest observed instances among the 5 types of human activities (see Table S1), but its negative impact on squirrels’ innovation performance was similar to dog-walking as discussed above. The significance of playground activity may affect species-specific predator responses. For example, tree squirrels such as the current study species use interspecific and intraspecific calls for threat detection (Randler 2006). Anthropogenic noise, like that generated from playgrounds, may mask the detection of predators or reduce effectiveness in communication, which leads to acute impacts on wildlife behavior (Blickley and Patricelli 2010). In our case, playground noise likely disturbed squirrels when they were attempting to solve novel problems on the ground.

The fact that different human activities varied their effect size on innovative problem-solving performance suggests that some squirrels may be able to differentiate between different types of human activities in their risk assessment, which has been shown in other tree squirrels species such as fox squirrels, *S. niger*, residing in urban areas differentiating the sound of humans and natural predators (Kittendorf and Dantzer 2021). Urban squirrels also show inter-individual differences in their response to human intensity and presence (Shuttleworth 2000; Krauze-Gryz et al. 2021). More investigations could be conducted in this area, such as identifying the cues that these urban red squirrels may have been using to differentiate human activities as well as the relationship between consistent individual differences in risk sensitivity and innovative problem-solving performance.

Increased distance to the nearest footpath decreased squirrels’ success rate, which contradicted our expectations. This result may be explained by a perceived higher threat further from the footpath. Our observations indicate that two-thirds of dog walkers (average 70.6% across all sites) disregarded the law that dogs must be kept on a leash in public areas (Järjestyslaki 27.6.2003/612, Section 14, the Finnish Public Order Act) and that humans (and their dogs) can walk freely through the woods. Therefore, being nearer to the footpath can increase visibility for a squirrel to detect these potential stressors sooner. Our analyses here did not explore interaction effects due to limited sample size. However, the role of distance to the nearest footpath in innovative problem-solving performance is likely partially independent, which may be related to the intertwined characteristics in urban environments (Boeing 2018). That is, footpaths may share some variance with other factors (eg, number and types of human activity), leading to a lower detection of the significance of either factor (eg, footpath or cycling) on solving performance.

Our field experiment demonstrates that an increased number of humans in a site and activities, most notably walking and dog-walking, significantly impair Eurasian red squirrels’ ability to solve a novel food-extraction task. Our findings suggest that these human activities may exert selective pressure on shaping wildlife cognition, and thereby affect their ability to adapt to urban environments (Sih 2013). Importantly, our results concerning the negative effects of these human activities on squirrel cognition may be useful for evaluating or tightening existing urban management and policy, such as leash laws or pet-free zones around key foraging sites, which could alleviate stress on urban squirrels (and other wild animals).

## Supplementary Material

araf104_Supplementary_Data

## Data Availability

Analyses reported in this article can be reproduced using the data provided by Author (2025).

## Pre-print in BioRvix

https://biorxiv.org/cgi/content/short/2024.11.20.624604v1.
