# Supplementary material for: Impact of human presence and activity on urban Eurasian red squirrels’ innovative problem-solving
Source: Behav Ecol. 2025 Sep 16;36(5):araf104. doi: 10.1093/beheco/araf104 (PMC12527259; doi:10.1093/beheco/araf104)
Supplement: araf104_Supplementary_Data [file araf104_supplementary_data.zip › SI_Chow, Loukola & Solvi_R2.docx]

Supplementary information

**Impact of Human Presence and Activity on Urban Eurasian Red Squirrels' Innovative Problem-Solving**

This document contains additional notes, tables, figures, and links to videos of the main manuscript.

Note S1-S2

Table S1-S6

Video S1-S2

Note S1 Geographic information, habitat type, of the 15 field sites (Oulu, Finland) on Google Earth

<https://earth.google.com/earth/d/1vHisjzF8p4AtRtrLEIfn-ANh4tymtODG?usp=sharing>. Also see Table S1

Note S2. Individual identification

To identify each individual and also record the number of squirrels in each site, we used an established method by Chow et al., (2018) using frame-by-frame analysis using Adobe Premiere Pro CS6. The first identification required intensive observer training that lasted for two months. This analysis is similar to the ‘mark-recapture’ and ‘mark-resight’ methods but on video footage. When we saw a squirrel appear on a video for the first time, it was ‘marked’ using their characteristics. Each squirrel was assigned a name and an identification number. This process required back and forth watching different footage so that the individuals’ full characteristics could be revealed from different angles. It was ‘recaptured’ when it reappeared in the subsequent videos. We recorded detailed characteristics of each squirrel that included their facial marking (e.g. a white dot/patch on face), colouration (e.g. orange, burgundy, brown patch on forehead), the colour of their limbs (e.g. orange/dark brown paws or dots on a toe) alongside height (relative to the apparatus), tail and body shape (e.g. full fur tail, half tail). We reidentified the squirrels three to five months after the first identification during which the same coder (the first author, PKYC) re-conducted the frame-by-frame analyses of the unmarked individuals. To examine the agreement between the two-time measures of the same coder, we ran an intra-rater reliability test using Cohen’s Kappa (Kappa = 0.99).

Table S1. Information about each site (N = 15). Each site represents a district or a subsection of a district within the city of Oulu, Finland. In each site, we selected one location for one puzzle box. Although the exact spatial extent of each site could not be formally defined due to the absence of official boundaries, our site classifications followed the designations of the National Land Survey of Finland (NLS Finland). Except two locations that were ~300m apart (indicated as ‘*’ in the table), all sites were approximately 400–500 meters apart to minimise the likelihood of sampling the same individuals at multiple sites (this is verified by video analyses see Note S2). The table includes the site name, classification of the site (data from [NLS Finland](https://www.maanmittauslaitos.fi/en/e-services/geodata-portal-paikkatietoikkuna)), location type, geographic coordinates (data collected from [Google Earth](https://earth.google.com/web/@65.02905473,25.46434192,18.97917306a,10157.65719416d,30.37022697y,0h,0t,0r/data=CgRCAggBMikKJwolCiExdkhpc2p6RjhwNEF0UnRyTEVJZm4tQU5oNHR5bXRPREcgAToDCgEwQgIIAEoHCLrL0zkQAQ)), distance to the nearest footpath in meters (m), the total number of records (observations) made throughout the field experiment, and the number of observations for each type of human activity in percentage (i.e., the number of observations of each type of activity divided by the total number of observations across the field experimental day in a site). Types of activities include walking, dog walking, cycling, playground activities, and other activities (e.g., bin collection, grass cutting, exercising, and cross-country skiing).

| Site name | Classification of location | Location type | Geographic  Coordinates | Squirrel group size | Distance between the location of the box to the nearest footpath (m) |
| --- | --- | --- | --- | --- | --- |
| Niittyaro | Quarter | Fragmented forest | 65°02'30"N 25°27'00"E | 4 | 19.65 |
| Vanhakangas | Quarter | Urban park | 65°02'38"N 25°26'21"E | 3 | 19.10 |
| Toppila | District | Fragmented forest | 65°02'51"N 25°26'05"E | 3 | 17.89 |
| Taskila | District | Fragmented forest | 65°03'05"N 25°25'40"E | 4 | 79.39 |
| Tuira | District | Urban park | 65°01'28"N 25°27'47"E | 5 | 45.03 |
| Myllytulli -Ainolan puisto | District | Urban park | 65°00'58"N 25°28'32"E | 6 | 36.32 |
| Raskila | District | Urban park | 65°00'36"N 25°30'01"E | 3 | 23.10 |
| Karjasilta - Uma | District | Urban park | 65°00'14"N 25°29'40"E | 4 | 14.93 |
| Välivainio | District | Fragmented forest | 65°02'12"N 25°28'18"E | 4 | 25.43 |
| Alppila | District | Fragmented forest | 65°02'04"N 25°27'23"E | 4 | 20.50 |
| Puolivälinkangas | District | Fragmented forest | 65°02'29"N 25°29'15"E | 4 | 17.66 |
| Isko | District | Fragmented forest | 65°02'47"N 25°28'12"E | 4 | 59.47 |
| Koskela | District | Fragmented forest | 65°02'49"N 25°27'30"E | 3 | 35.63 |
| Syynimaa* | Quarter | Fragmented forest | 65°03'01"N 25°27'36"E | 3 | 50.88 |
| Linnanmaa* | District | Fragmented forest | 65°03'07"N 25°27'51"E | 5 | 25.48 |

Table continue…

| District  Site name | Total number of records | Types of human activity | | | | | | |
| --- | --- | --- | --- | --- | --- | --- | --- | --- |
|  |  | Walking (%) | Dog walking (%) | Cycling (%) | Playground activities (%) | Other activities (%) | Walking (%) | Dog walking (%) |
| Niittyaro | 88 | 11.4 | 6.8 | 8.0 | 0.0 | 0.0 | 11.4 | 6.8 |
| Venhopuisto | 86 | 29.1 | 24.4 | 29.1 | 5.8 | 8.1 | 29.1 | 24.4 |
| Toppila | 81 | 25.9 | 14.8 | 29.6 | 0.0 | 7.4 | 25.9 | 14.8 |
| Taskila | 78 | 15.4 | 7.7 | 9.0 | 0.0 | 5.1 | 15.4 | 7.7 |
| Tuira | 73 | 39.4 | 13.6 | 1.5 | 0.0 | 0.0 | 39.4 | 13.6 |
| Myllytulli -Ainolan puisto | 86 | 82.6 | 18.6 | 82.6 | 0.0 | 23.3 | 82.6 | 18.6 |
| Raskila | 73 | 61.6 | 13.7 | 38.4 | 0.0 | 2.7 | 61.6 | 13.7 |
| Karjasilta - Uma | 80 | 16.3 | 10.0 | 15.0 | 27.5 | 2.5 | 16.3 | 10.0 |
| Välivainio | 69 | 13.0 | 14.5 | 0.0 | 0.0 | 1.4 | 13.0 | 14.5 |
| Alppila | 69 | 71.0 | 36.2 | 46.4 | 5.8 | 0.0 | 71.0 | 36.2 |
| Puolivälinkangas | 73 | 50.7 | 20.5 | 43.8 | 0.0 | 0.0 | 50.7 | 20.5 |
| Isko | 64 | 3.1 | 1.6 | 0.0 | 0.0 | 0.0 | 3.1 | 1.6 |
| Koskela | 79 | 88.6 | 35.4 | 41.8 | 0.0 | 30.4 | 88.6 | 35.4 |
| Syynimaa* | 76 | 9.2 | 5.3 | 0.0 | 0.0 | 1.3 | 9.2 | 5.3 |
| Linnanmaa* | 73 | 39.7 | 28.8 | 50.7 | 16.4 | 1.4 | 39.7 | 28.8 |

Table S2. Model specification. This table includes the fixed variable, model distribution, response variables (at the top) and random variables (in the box) for each model.

|  | | Response variables | | | | |
| --- | --- | --- | --- | --- | --- | --- |
|  |  | (a) Site level (N = 15): Proportion of first-visit success (first-visit solvers) | (b) Site level (N = 15): Proportion of first and subsequent visit success (all solvers) | (c) individual level (N = 64): success outcome on first and subsequent visit | (d) individual level (N = 43): first success latency for first-visit solvers | (e) individual level (N = 53): first-success latency for all solvers |
| M | Fixed factors/distribution | Beta regression | Beta regression | Binomial | Gamma | Gamma |
| 1 | Squirrel group size | N/A | N/A | Site | Site | Site |
|  | Mean number of humans present per observation |  |  |  |  |  |
|  | Distance of nearest footpath |  |  |  |  |  |
| 2 | Walking | N/A | N/A | Site | Site | Site |
|  | Dog walking |  |  |  |  |  |
|  | Playground activities |  |  |  |  |  |
|  | Distance of nearest footpath |  |  |  |  |  |
| 3 | Cycling | N/A | N/A | Site | Site | Site |
|  | Dog walking |  |  |  |  |  |
|  | Playground activities |  |  |  |  |  |
|  | Distance of nearest footpath |  |  |  |  |  |

Table S3. Site-level analysis (proportion of success) (N = 15). Correlations between fixed variables, mean human presence nearby, squirrel group size and distance to the nearest footpath. Human presence nearby was the mean number of humans present within a 100 m radius around the puzzle box during each check. Squirrel group size was the number of squirrels that participated in the experiment. Distance to the nearest footpath (m) was the shortest distance between the puzzle box and the nearest footpath.

|  | Squirrel group size | Mean human presence nearby |
| --- | --- | --- |
| Mean human presence nearby | 0.329 |  |
| Distance to nearest footpath | 0.266 | -0.176 |

Table S4. Site-level analysis (proportion of success) (N = 15). Correlations between types of human activity (walking, dog walking, cycling, and playground activity) and distance to the nearest footpath (m). To avoid multicollinearity, variables with Pearson correlation r ≥ 0.5 (bold values) are not included in the same model during analyses.

|  | Walking | Dog walking | Cycling | Playground activity |
| --- | --- | --- | --- | --- |
| Dog walking | 0.35 |  |  |  |
| Cycling | **0.94** | 0.31 |  |  |
| Playground activity | -0.20 | 0.01 | -0.09 |  |
| Distance to the nearest footpath | -0.07 | -0.41 | -0.12 | -0.31 |

Table S5. Individual-level analysis (first-success latency). Only first and subsequent solvers provided information about solving latency to obtain first success (N = 53). Correlations between fixed variables, mean human presence nearby, squirrel group size and distance to the nearest footpath. Human presence nearby was the mean number of humans present within a 100 m radius around the puzzle box during each check. Squirrel group size was the number of squirrels that participated in the experiment. Distance to the nearest footpath (m) was the shortest distance between the puzzle box and the nearest footpath.

|  | Squirrel group size | Mean human presence nearby |
| --- | --- | --- |
| Mean human presence nearby | 0.39 |  |
| Distance to nearest footpath | 0.22 | -0.13 |

Table S6. Individual-level analysis (first-success latency). Only first and subsequent solvers provided information about solving latency to obtain first success (N = 53). Correlations between types of human activity (walking, dog walking, cycling, and playground activity) and distance to the nearest footpath (m). To avoid multicollinearity, variables with Pearson correlation r ≥ 0.5 (bold values) are not included in the same model during analyses.

|  | Walking | Dog Walking | Cycling | Playground activity |
| --- | --- | --- | --- | --- |
| Dog Walking | 0.33 |  |  |  |
| Cycling | **0.95** | 0.31 |  |  |
| Playground activity | -0.21 | 0.07 | -0.1 |  |
| Distance to the nearest footpath | -0.02 | -0.43 | -0.08 | -0.33 |

Supplementary video

[S1.](https://osf.io/s87kc) An innovator, Panda, pushed a lever-end to solve the novel problem. This if it is close to a nut container, or pull (instead of push) the lever-end if it is far from the nut container so as to make a lever/nut drop (i.e. successful solving).

demonstrated the solutions for this problem are counter-intuitive to squirrels in which a squirrel (demonstrated by Mario here; also see video ‘PST’ in the electronic supplementary material)

[S2.](https://osf.io/qzu8a) A squirrel’s response to approaching humans while she was solving the task.
